# The Comparison of the Anti-inflammatory Efficacy of Phytochemical Extracts in Punica granatum and Lawsonia inermis Among Patients Diagnosed With Chronic Periodontitis

**DOI:** 10.7759/cureus.47557

**Published:** 2023-10-24

**Authors:** Bharani Krishna Takkella, Nayanala Venkata Anusha, D Lokanathan Balaji, MP Venkata Prabhat, Gummadapu Sarat, Tejaswin Polepalle, Mohammad Naffizuddin, Chukka Ramsunil, Varri Sujana, Thakkella Chaitanya Krishna

**Affiliations:** 1 Oral Medicine and Radiology, Drs. Sudha and Nageswara Rao Siddhartha Institute of Dental Sciences, Vijayawada, IND; 2 Prosthodontics, Priyadarshini Dental College and Hospital, Thiruvallur, IND; 3 Periodontology, Faculty of Dentistry, Malaysian Allied Health Sciences Academy (MAHSA) University, Jenjarom, MYS; 4 Oral and Maxillofacial Surgery, Care Dental College and Hospital, Guntur, IND; 5 Conservative Dentistry and Endodontics, Sibar Institute of Dental Sciences, Guntur, IND; 6 Mechanical Engineering, Teesside University, Middlesbrough, GBR

**Keywords:** aspartate aminotransferase (ast), alanine aminotransferase (alt), lactate dehydrogenase (ldh), infrared spectrophotoscopy (ifsc), interquartile range (iqr), phytochemicals, lawsonia inermis, punica granatum, periodontitis, inflammation

## Abstract

Introduction

The oral cavity is the gateway to the human body. Periodontitis is a common inflammatory condition affecting the oral cavity and a known etiological cause of tissue destruction, discomfort, and halitosis. Pomegranate (*Punica granatum*) and henna (*Lawsonia inermis*) are herbs known to mankind from time immemorial whose extracts are proven to fight inflammation. The current study was done to evaluate the phytochemical anti-inflammatory efficacy of *Punica* and *Lawsonia* in patients with chronic periodontitis and test the potency of herbal mouthwashes in fighting the inflammatory condition affecting the oral cavity using distilled water as a control group.

Materials and methods

A double-blinded randomized control trial was conducted on 60 patients who were recruited and divided into three groups, in which 20 patients were prescribed with pomegranate (*Punica*: n=20) mouthwash and 20 patients with henna (*Lawsonia*: n=20) mouthwash along with distilled water (n=20). All patients were randomly allocated using the coin toss method and advised to use the prescribed mouthwash for a period of two weeks. Unstimulated saliva was collected before using the mouthwash, and salivary enzymes such as aspartate aminotransferase (AST), alanine aminotransferase (ALT), and lactate dehydrogenase (LDH) and their levels were assessed spectrometrically using the infrared spectrophotoscopy (IFSC) method. Each patient was assigned a mouthwash and recalled after two weeks. Unstimulated saliva was again collected, and salivary activity levels of enzymes AST, ALT, and LDH were analyzed after using mouthwash in a similar method as done before. Later on, the salivary levels of enzymes AST, ALT, and LDH were compared before and after the usage of mouthwashes. Statistical significance was seen in the salivary enzymatic activity of AST, ALT, and LDH before and after using *Punica* and *Lawsonia* mouthwashes due to their potent phytochemical action in fighting inflammation. Statistical analysis was performed using Statistical Package for Social Sciences (SPSS) 22 (IBM SPSS Statistics, Armonk, NY). The Shapiro-Wilk test was used to determine the normality and significance; intragroup comparison was done using the Wilcoxon signed-rank test and Mann-Whitney U test. Intergroup comparison was done using the Kruskal-Wallis test.

Results

*Punica* patients had much lower levels of salivary AST and ALT (p<0.001) and a decrease in LDH (p=0.002) after the usage of mouthwash for a period of two weeks. Also, patients using *Lawsonia* as herbal mouthwash had reduction in the values of AST (p=0.001) and LDH (p=0.003) and prominent reduction in ALT (p<0.001) after a period of two weeks. But in the case of patients using distilled water, there was an increase in the salivary enzymatic activity of AST and ALT, which was statistically significant (p<0.001), and LDH (p=0.006) depicting the disease progression even after using mouthwash for the given time period of two weeks.

Conclusion

This study demonstrated that both *Punica *and *Lawsonia* were effective in reducing the inflammation in patients diagnosed with chronic periodontitis. However, when intergroup comparison was done, the anti-inflammatory efficacy was superior in* Punica* with significant reduction in the parameters such as of AST, ALT, and LDH when compared to *Lawsonia *owing to its potent phytochemical constituency in cutting down the inflammation. Hence, *Punica *can be used as an implicated effective anti-inflammatory herbal mouthwash.

## Introduction

Inflammation, a "double-edged sword," is known for its genuine nature of being helpful and sometimes also harmful to the human body based on many clinical observations. Clinically, inflammation is characterized by five cardinal signs: redness (rubor), swelling (tumor), heat (calor, only applicable to the body extremities), pain (dolor), and loss of function (functio laesa). These were the signs given by Celsus in ancient Rome (30-38 BC) and then added to by Galen (AD 130-200) as described by Todorovic et al. [1].

Inflammation is "the succession of changes that occur in a living tissue when it is injured provided that the injury is not of such a degree as to at once destroy its structure and vitality" or "the reaction to injury of the living microcirculation and related tissues." Even though in ancient times inflammation was recognized as being a part of the healing process, until the beginning of the 20th century, inflammation was viewed as an undesirable response to the host. Research to date reveals that the major risk factors for many diseases are infections, tobacco, alcohol, radiation, obesity, environmental pollutants, and diet. These factors are proven to drive chronic diseases through the induction of inflammation.

Inflammation is of two types: acute and chronic. The initial acute inflammation is a physiological response to the microbial challenge for recruiting cells to the site of infection through inflammatory mediators, whereas chronic inflammation persists for a long time, owing to many morbid diseases such as cardiovascular, neurodegenerative, and respiratory diseases. Prasad and Kunnaiah suggested that the aforementioned risk factors induced cancer through chronic inflammation [2].

Mouthwashes play an important role in maintaining oral hygiene. However, conventional mouthwashes currently available in the market contain chlorhexidine, which is known to increase the risk of toxicity and staining of teeth and altered microflora in the oral environment for the general population. "The current study examines the efficacy of herbal mouthwashes in fighting periodontitis. They contain potent phytochemicals that fight inflammation and induce fewer side effects than chemical mouthwashes. The only known side effect is allergy to the particular herb used in the preparation of mouthwash, which we could not find in our study. The bioavailability of these phytochemicals has been increased in newer generations of herbal mouthwashes." Herbal mouthwash provides a viable alternative as they are alcohol-free and chemical-free and contain time-tested herbal oils and extracts such as neem oil, clove, and peelu that actually promote oral health.

Periodontitis is a chronic inflammatory condition of the periodontium in the advanced stage during which the expansion of the periodontal ligament and the obliteration of the alveolar bone may be observed. It is one of the two leading hazards to oral health and the primary cause of tooth loss as described by Bhavana et al. [3]. The oral cavity contains over 800 distinct species of bacteria, and periodontal disease can be caused by a complex combination of bacterial infection and host response, influenced by behavioral variables such as smoking and local factors such as calculus and poor dental hygiene [3]. One of the known common etiological factors of chronic periodontitis is poor oral hygiene and the lack of interdental cleansing devices, which leads to the accumulation of supragingival and subgingival plaques, which in turn leads to the deposition of calculus. The widely known periodontal pathogens present in plaque include aerobic and anaerobic bacteria such as *Streptococci* species, *Porphyromonas gingivalis*, *Prevotella intermedia*, *Treponema denticola*, *Campylobacter rectus*, *Selenomonas* species, *Eubacterium timidum*, *Fusobacterium nucleatum*, and *Parvimonas micra*. Jeffcoat et al. [4] have designated three species as chief etiological agents of periodontitis, namely, *Actinobacillus actinomycetemcomitans*, *Porphyromonas gingivalis*, and *Tannerella forsythia*; these organisms were responsible for periodontal destruction. Herbal mouthwashes in the near future may serve better compared to conventional mouthwashes. However, futuristic clinical trials are more required to bring out evidence-based herbal mouthwashes. The hectic lifestyle that most people lead results in infrequent visits to a dentist for the removal of calculus. Hence, chemical alternatives such as mouthwash are used to fight inflammation and maintain oral hygiene. However, they are associated with several side effects, including staining and a burning sensation.

Mother Nature has bestowed on us a plethora of treatments for many different diseases. Phytochemicals are now emerging as innovative medications with ongoing drug clinical studies in several disorders of the oral cavity. In their natural and synthetic forms, they have long been utilized to treat a variety of chronic diseases. Because they have fewer side effects, herbal mouthwashes are becoming more and more popular, allowing us to prolong their therapeutic effect to reduce the incidence of the common inflammatory condition affecting the oral cavity as described by Rao et al. [5].

Plants in the Lythraceae family have been shown to have high medicinal properties. *Punica *and *Lawsonia* have also demonstrated effective phytomedicinal properties [5]. *Punica granatum*, commonly known as pomegranate, is a tropical fruit well known for its taste and medicinal benefits attributed to its phytochemical constituents such as punicalin, phenols, and triterpenoids. Here, the dried and powdered exocarp of the *Punica granatum* fruit is taken. *Lawsonia inermis*, commonly known as henna, is an annual herb belonging to Lythraceae that is mainly used for ornamental purposes by females on various occasions [5]. In this study, a leafy extract of *Lawsonia inermis* was used in a mouthwash preparation. Aside from that, it has various medicinal properties owing to its phytochemical constituents, such as 2-hydroxy naphthoquinone, saponins, and xanthones, which have antimicrobial properties [3]. In this study, the anti-inflammatory efficacy of *Punica* and* Lawsonia* was compared with the normal control groups, who were given distilled water as a mouthwash.

## Materials and methods

The study was carried out in the Department of Oral Medicine and Radiology at Drs. Sudha and Nageswara Rao Siddhartha Institute of Dental Sciences in Chinnaoutpalli, Vijayawada, Andhra Pradesh, India, from January to March 2023. *Punica granatum *and *Lawsonia inermis*, two plants of the Lythraceae family, were tested for their ability to reduce inflammation in individuals with chronic periodontitis. The current study's procedures were carried out as per the 2019 Helsinki Declaration. The institute's Institutional Ethical Committee (IEC) approved the study, bearing IEC number O.C.No./IEC/52F-2023.

A total of 72 patients were selected for the current double-blinded randomized control study, of which 12 patients were not willing to participate, so this study included 60 patients between the ages of 40 and 70. These patients were divided into three groups. Informed consent was taken from the patients before starting the study procedures. In Group I, 20 patients were given *Punica* mouthwash; in Group II, 20 patients were given *Lawsonia* mouthwash; and in Group III, 20 patients were given distilled water as a mouthwash.

The recommendations conformed with those of the ethical committee. Patients visiting the dental outpatient clinic provided their informed consent before being identified as having chronic generalized periodontitis based on the clinical and radiographic evaluation. A total of 60 patients were chosen, and those with clinical attachment loss of more than 3 mm and periodontal probing depths of between 5 and 7 mm were considered to have the disease. Russell's periodontal index evaluation parameters based on probing depth and clinical attachment loss were used to categorize the patients. A single dentist performed each examination to standardize things, and another dentist double-checked the work. Aspartate aminotransferase (AST), alanine aminotransferase (ALT), and lactate dehydrogenase (LDH) were the salivary enzymes used as inflammatory mediators to see before and after the use of mouthwash.

Exclusion criteria

Patients with uncontrolled diabetes mellitus; a history of aggressive periodontitis, autoimmune diseases, corticosteroid therapy, smoking, chronic alcoholism; a previous history of periodontal therapy, including scaling for a time period of less than six months; and previous histories of malignancy were excluded from the study.

Preparation of crude mouthwash

The dried bark of a wild *Punica* fruit and *Lawsonia* leaves were collected from Heena Industries and Pure Agri Tech in January 2023. The dried leaves of* Lawsonia* along with the dried bark of* Punica* were later ground up to mesh size 42 and then pulverized into a fine powder separately using the method described by Bhavana et al. [3].

Plant extract

**Figure 1:**
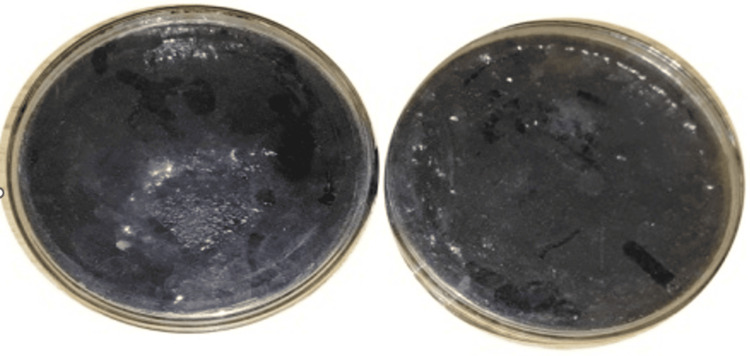
Liquid extract of Punica and Lawsonia

First, a precision balance was used to weigh the raw material, and then, ethanol and chloroform were used as solvents during the extraction process. Ten grams of powder bark from *Punica* and leaf from *Lawsonia* were mixed with 100 mL of ethanol and shaken for three hours in a rotary shaker at a speed of 180 revolutions per minute (rpm) until broken down. Subsequently, size 1 Whatman filter paper was used to filter the residue. The resulting residue was dried at 60°C in a rotatory evaporator under decreased pressure (Figure 1).

Phytochemical analysis

For standard phytochemical screening procedures, the obtained extracts were put through qualitative phytochemical analysis to check for the presence of specific phytochemical components, such as naphthoquinones, alcohols, steroids, xanthones, coumarins, and flavonoids for *Lawsonia* and amino acids, proteins, flavonoids, tannins, steroids, saponins, carbohydrates, alkaloids, and glycosides for *Punica* [3,5-8].

Preparation of mouthwash

The dried leaves were initially ground to fine particles of mesh size 42 and later on made into a fine powder using a blender. Initially, the raw material was taken in a precision balance, and then, the extraction procedure was done using ethanol and chloroform as solvents. The final yield concentrate was approximately 0.8 g for each extract, which was then superadded with glycerol 10 mL, followed by 0.2 g L-menthol, 0.005 g peppermint oil, and 0.2 g citric acid. To this resultant mixture, distilled water was added to make a solution of 100 mL, which was then transferred to glass bottles and stored separately for *Lawsonia *and* Punica* [3].

Study procedure

Selected subjects and the examiner were blinded to allocate the product. Groups I, II, and III were assigned mouthwashes separately. Patients were advised to use their assigned mouthwash, followed by their routine toothbrushing. Before the use of their assigned mouthwash, patients were asked to rinse the oral cavity thoroughly with water once and then with physiological saline to remove any accumulated debris from the oral cavity between 10:00 am and 12:00 pm to avoid diurnal variation [3].

Collection of saliva at baseline

Later, unstimulated saliva was collected by the spitting method (i.e., asking the subjects to keep their heads downward to allow for the pooling of saliva in the oral cavity completely until the patient feels the urge to spit or swallow). At this point, 3-5 mL of saliva was collected in a separate small bottle with markings. After 15 minutes, the patients were asked to use their assigned mouthwash in front of the dentist and were made to sit for one hour to check for any allergic reaction. But we could not find any.

Standard regimen

After saliva was collected from each patient, patients were assigned a particular type of mouthwash, and a standard regimen of 5 mL per rinse twice a day was advised to all the recruited subjects using the mouthwash for a period of two weeks. Patients were also advised to maintain a diary for two weeks to mark the usage of mouthwash twice daily without any mishaps. They also received follow-up phone calls to remind them about the usage of mouthwash.

Method for the estimation of AST, ALT, and LDH

The collected saliva was later transferred to a micropipette and transferred to a test tube that was later subjected to centrifugation at 10,000 rpm for 10 minutes.

To determine the activity of salivary enzymes AST, ALT, and LDH, the saliva in the centrifuged test tubes was spectrometrically analyzed using the infrared spectrophotoscopy (IFSC) method on a Mindray (Shenzhen, China) automatic analyzer.

Collection of saliva after two weeks

After two weeks, each patient was recalled, and again, unstimulated saliva was collected for which the enzymatic activity of AST, ALT, and LDH as analyzed, likewise as it was done previously at baseline level, and the values were noted for each group of patient separately, which is double-checked by another dentist.

## Results

Statistical analysis

The analysis was performed using the Statistical Package for Social Sciences (SPSS) 22 (IBM SPSS Statistics, Armonk, NY). The quantitative variables were expressed as mean and standard deviation along with median and interquartile range (IQR). The Shapiro-Wilk test was used to determine the normality and also to calculate the significance; intragroup comparison was done using the Wilcoxon signed-rank test and Mann-Whitney U test. Intergroup comparison was done using the Kruskal-Wallis test. Statistical significance was set at p<0.05, which was considered a significant association at a 5% level of significance, whereas p<0.001 was considered highly significant, and p>0.05 was considered as not significant.

Results

The numerical values of AST and ALT in the intragroup comparison were increased after using distilled water, and a highly significant p<0.001 was obtained​​. Similarly, LDH values were increased after using distilled water mouthwash with statistical significance (p=0.006) as observed in Table 1.

**Table 1:** Intragroup comparison of AST, ALT, and LDH before and after using distilled water mouthwash SD, standard deviation; IQR, interquartile range; AST, aspartate aminotransferase; ALT, alanine aminotransferase; LDH, lactate dehydrogenase

Group	Variable	Mean	SD	Median	IQR	Minimum	Maximum	p-value
Distilled water	AST before	103.2	60.8	85.4	64.1	15.1	261.2	<0.001
	AST after	114.4	64.2	106.0	80.7	23.1	258.8	
	ALT before	45.0	34.5	38.1	58.7	1.2	111.8	<0.001
	ALT after	56.2	37.9	49.3	76.5	1.8	118.2	
	LDH before	317.4	195.2	294.5	186.3	119.2	948.0	0.006
	LDH after	353.4	200.2	305.4	186.9	138.0	1,019.0	

In the intragroup comparison, the numerical values of AST, ALT, and LDH was reduced after using* Punica* mouthwash. There was a highly significant reduction in the AST and ALT with p<0.001. But in the case of LDH, there was a significant reduction after the usage of mouthwash with p=0.002. The reduction of LDH was not pronounced as much as in the case of AST and ALT for *Punica* mouthwash as seen in Table 2.

**Table 2:** Intragroup comparison of AST, ALT, and LDH before and after using Punica mouthwash SD, standard deviation; IQR, interquartile range; AST, aspartate aminotransferase; ALT, alanine aminotransferase; LDH, lactate dehydrogenase

Group	Variable	Mean	SD	Median	IQR	Minimum	Maximum	p-value
Punica	AST before	109.9	61.9	93.1	67.7	14.5	283.4	<0.001
	AST after	27.3	18.8	25.1	22.3	4.1	74.6	
	ALT before	36.0	35.8	20.1	49.9	2.1	118.6	<0.001
	ALT after	22.6	27.8	6.7	32.5	1.4	98.2	
	LDH before	354.4	222.4	296.5	241.1	59.3	947.0	0.002
	LDH after	84.6	48.3	77.3	58.8	19.0	184.8	

The numerical values of AST, ALT, and LDH in the intragroup comparison were reduced before and after using* Lawsonia* mouthwash. There was significance in the reduction of AST and LDH with p=0.001 and p=0.003, respectively. But in the case of ALT, there was a highly statistical significance in the reduction before and after using mouthwash with p<0.001. The reduction of AST and LDH was not stressed as much as that of ALT in the case of *Lawsonia* mouthwash as depicted in Table 3.

**Table 3:** Intragroup comparison of AST, ALT, and LDH before and after using Lawsonia mouthwash SD, standard deviation; IQR, interquartile range; AST, aspartate aminotransferase; ALT, alanine aminotransferase; LDH, lactate dehydrogenase

Group	Variable	Mean	SD	Median	IQR	Minimum	Maximum	p-value
Lawsonia	AST before	76.9	43.8	75.1	63.7	12.0	186.6	0.001
	AST after	35.1	24.1	26.5	35.8	4.5	86.4	
	ALT before	34.4	27.4	33.0	37.9	2.0	98.7	<0.001
	ALT after	22.7	21.5	17.0	34.8	1.8	81.4	
	LDH before	353.4	218.3	297.5	215.5	112.7	1,012.0	0.003
	LDH after	155.0	54.4	142.6	62.4	78.6	288.3	

When intergroup comparison was done for AST between the three groups before the use of mouthwashes, no statistically significant difference could be observed with p=0.11. Although after the use of mouthwashes, significant difference could be seen in the AST levels with p<0.01. Similarly, when the intergroup comparisons were made between the mouthwashes, a highly significant difference could be observed between *Lawsonia* and distilled water with p=0.001, which indicates the drastic reduction in the inflammatory component between the two groups (Table 4).

**Table 4:** Intergroup comparison of AST before and after using mouthwashes SD, standard deviation; IQR, interquartile range; AST, aspartate aminotransferase

Variable	Group	Mean	SD	Median	IQR	Minimum	Maximum	p-value	*Punica* versus *Lawsonia*	*Punica* versus distilled water	*Lawsonia* versus distilled water
AST before	Punica	109.9	61.9	93.1	67.7	14.5	283.4	0.11	-	-	-
	Lawsonia	76.9	43.8	75.1	63.7	12.0	186.6				
	Distilled water	103.2	60.8	85.4	64.1	15.1	261.2				
AST after	Punica	27.3	18.8	25.1	22.3	4.1	74.6	<0.001	0.34	0.51	<0.001
	Lawsonia	35.1	24.1	26.5	35.8	4.5	86.4				
	Distilled water	114.4	64.2	106.0	80.7	23.1	258.8				

The intergroup comparison of ALT between all groups before using mouthwashes reveals that there was no statistical significance with p=0.62. But after using it for a given period of two weeks, there was a significant reduction in the level of ALT with p=0.002. Also, when intergroup comparison was done between* Punica *and *Lawsonia*, as well as *Lawsonia* and distilled water, this revealed a highly significant difference with p<0.001 depicting the decrease in the inflammation after the usage of mouthwashes (Table 5).

**Table 5:** Intergroup comparison of ALT before and after using mouthwashes SD, standard deviation; IQR, interquartile range; ALT, alanine aminotransferase

Variable	Group	Mean	SD	Median	IQR	Minimum	Maximum	p-value	*Punica* versus *Lawsonia*	*Punica* versus distilled water	*Lawsonia* versus distilled water
ALT before	Punica	36.0	35.8	20.1	49.9	2.1	118.6	0.62	-	-	-
	Lawsonia	34.4	27.4	33.0	37.9	2.0	98.7				
	Distilled water	45.0	34.5	38.1	58.7	1.2	111.8				
ALT after	Punica	22.6	27.8	6.7	32.5	1.4	98.2	0.002	<0.001	0.002	<0.001
	Lawsonia	22.7	21.5	17.0	34.8	1.8	81.4				
	Distilled water	56.2	37.9	49.3	76.5	1.8	118.2				

The intergroup comparison of LDH levels before using mouthwashes reveals that there was no statistical significance with p=0.79. And after using the mouthwashes for a given period of two weeks, there was a highly significant reduction in the values of LDH with p<0.001. On comparison between each mouthwash, a highly significant difference could be seen between *Punica* and *Lawsonia* and *Lawsonia* and distilled water with p<0.001, which interprets the decrease in the salivary enzyme activity owing to a decrease in inflammatory response (Table 6).

**Table 6:** Intergroup comparison of LDH before and after using mouthwashes SD, standard deviation; IQR, interquartile range; LDH, lactate dehydrogenase

Variable	Group	Mean	SD	Median	IQR	Minimum	Maximum	p-value	*Punica* versus *Lawsonia*	*Punica* versus distilled water	*Lawsonia* versus distilled water
LDH before	Punica	354.4	222.4	296.5	241.1	59.3	947.0	0.79	-	-	-
	Lawsonia	353.4	218.3	297.5	215.5	112.7	1,012.0				
	Distilled water	317.4	195.2	294.5	186.3	119.2	948.0				
LDH after	Punica	84.6	48.3	77.3	58.8	19.0	184.8	<0.001	<0.001	0.003	<0.001
	Lawsonia	155.0	54.4	142.6	62.4	78.6	288.3				
	Distilled water	353.4	200.2	305.4	186.9	138.0	1,019.0				

**Figure 2:**
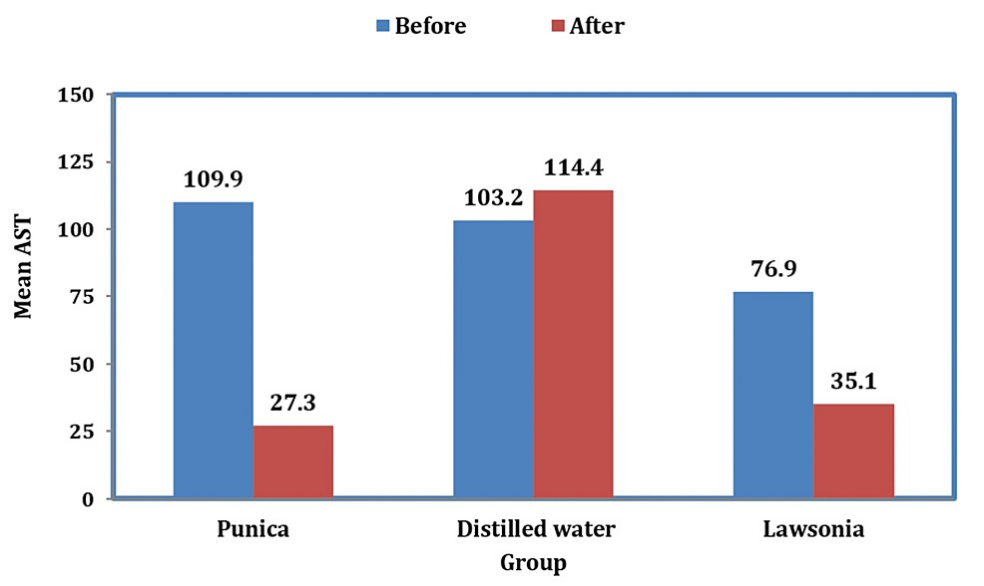
Graph representing the levels of AST before and after the usage of mouthwashes AST: aspartate aminotransferase

The AST levels were compared between all three groups where mean values were drastically reduced with *Punica* when compared to *Lawsonia* and no effect was seen with distilled water as the enzyme activity was increased (Figure 2).

**Figure 3:**
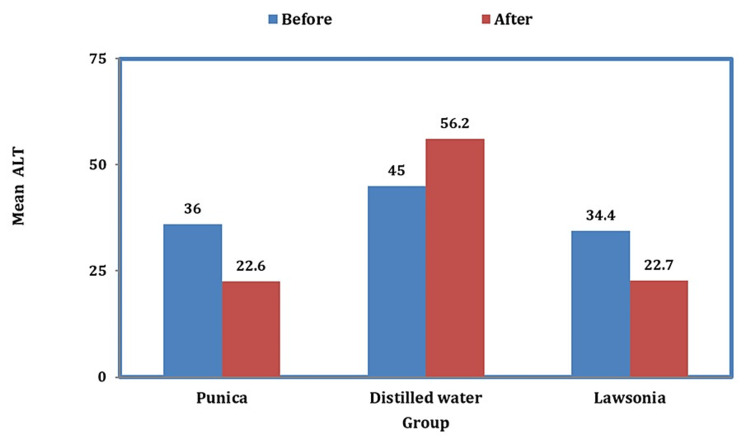
Graph representing the levels of ALT before and after the usage of mouthwashes ALT: alanine aminotransferase

When ALT levels were compared, there was a reduction before and after using mouthwash in *Punica* and similarly in the case of* Lawsonia*, but in the case of distilled water, there was an increase in the enzyme activity suggesting the continuation of disease progression (Figure 3).

**Figure 4:**
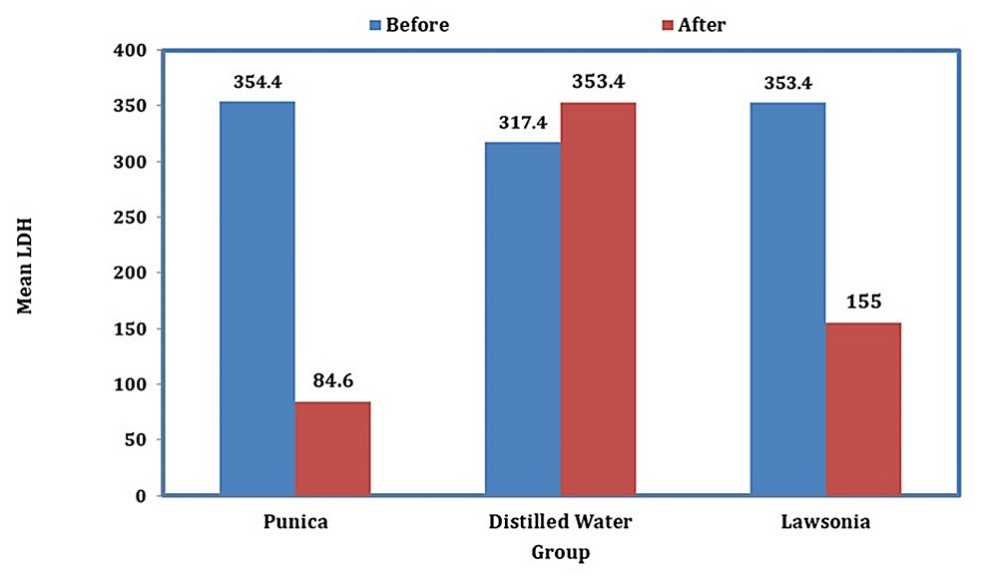
Graph representing the levels of LDH before and after the usage of mouthwashes LDH: lactate dehydrogenase

There was a radical reduction in the levels of LDH before and after using *Punica *mouthwash; likewise, a prominent reduction is seen even in the case of the *Lawsonia* group, but in the case of distilled water, the enzyme activity increased depicting no action in fighting the disease (Figure 4).

## Discussion

Saliva is recognized as a crucial biological fluid for the introduction of new diagnostic procedures that may aid in the diagnosis and treatment of a variety of novel systemic disorders. Among important salivary components, various enzymes are also released by stromal, epithelial, and bacterial cells in response to inflammation. Hassan et al. [9] and Sritrairat et al. [10] described that when bacteria attack, the host's defenses produce a response by increasing vascular permeability; increasing cellular response by increasing the number of polymorphonuclear neutrophils (PMNs), neutrophils, and monocytes; and releasing chemical mediators of inflammation including proinflammatory cytokines such as tumor necrosis factor-alpha (TNF-α) that cause an increase in the inflammatory response in the host, resulting in alterations in the extracellular matrix by decreasing collagen synthesis and increasing matrix metalloproteinase (MMP) activity, the proliferation of osteoclastic activity, and increases in tissue destruction.

Dabra et al. described how the diagnosis of many periodontal diseases usually relies on the gingival index, periodontal pocket depth, bleeding on probing tendency, and radiographic parameters [11]. Numerous markers have been proposed as diagnostic tests for periodontal disease, including intracellular enzymes. Various markers have been indicated as diagnostic aid markers of periodontal diseases [11]. According to Prakash et al., when periodontal tissue becomes diseased, the cells get destroyed through the activity of the host immune response or by chemical mediators released by bacterial cells. These damaged cells release enzymes that are increasingly released into the saliva and gingival crevicular fluid (GCF), which, in turn, results from their enhanced release from the periodontium's soft tissues and also reflects a shift in the metabolism of the inflamed gingiva [12].

The enzymes AST, ALT, and LDH belong to the category aspartate aminotransferase (AST) that takes part in the biochemical reaction L-aspartate+2-oxoglutarate in the presence of AST converted to oxaloacetate+L-glutamate, an important enzyme involved in amino acid metabolism and a potent marker of tissue damage. Alanine aminotransferase (ALT) takes part in the biochemical reaction L-alanine+2-oxoglutarate in the presence of ALT converted into pyruvate+L-glutamine, which is also an important step in amino acid metabolism and also assists in filtering toxins from the blood, storing iron and serving as a digestive aid. The biochemical reaction of lactate dehydrogenase (LDH) is LDH sample pyruvate+nicotinamide adenine dinucleotide plus hydrogen (NADH) in the presence of LDH converted into lactate+nicotinamide adenine dinucleotide (NAD+), a vital step in carbohydrate and fat metabolism. LDH is a potent marker of tissue damage, especially in cardiac muscle and skeletal muscle, as described by Lamba et al. [13]. The presence of these enzymes can be found in saliva as these activities were proven to be there, which was quite similar to their presence in blood. Krushna Kishore et al. documented that the increased activity of these enzymes in the saliva is probably due to the activity of destructive processes in the alveolar bone in advanced stages of the development of periodontal diseases [14].

In the current study, when distilled water was used as a mouthwash, the levels of enzymes AST and ALT were elevated with statistical significance, and LDH was not significant, indicating that the action of distilled water could not stop the disease progression, which resulted in the flare-up of inflammatory mediators. Phytochemicals are chemical constituents present in herbs that are responsible for their medicinal properties. The current study reveals that the composition of phytochemicals in *Punica* and *Lawsonia* was unique in fighting inflammation, which accords with the study conducted by Ahuja et al. [15].

A study done by Bhadbhade et al. focused on GCF, which always has a much closer connection to periodontal disorders and has implicated the presence of these enzymes. They mentioned that while GCF is a powerful marker of periodontal disease, collecting it presents significant challenges for both the patient and the observer. They concluded that using *Punica *as mouthwash causes a statistically significant decrease in salivary enzymatic activity [16]. Garachh et al. documented that *Punica granatum* has wound-healing properties [17]. Sreekumar et al. mentioned that *Punica granatum* can induce fibroblast proliferation and the formation of collagen and angiogenesis [18]. The antimicrobial action of *Punica* also plays a key role in reducing inflammatory markers by inhibiting the quorum sensing of periodontal microorganisms as reported by Triveni et al. [19].

In this study, *Lawsonia* decreased the activity of the enzymes AST and ALT, and the levels of LDH were reduced after the use of it as mouthwash with statistical significance. This action was attributed to the presence of phytochemicals in *Lawsonia* such as 1,4-naphthoquinone, xanthones, and saponins, which have anti-inflammatory effects [6]. But when *Punica* and *Lawsonia* were compared, much reduction is seen in the case of *Punica granatum*,which might be due to its chemical nature and potent phytochemical constituency.According to Deshmukh et al., phytochemicals such as gallic acid in *Punica* and 1,4-naphthoquinone in *Lawsonia *were responsible for the inhibitory action of the *Streptococcus* species [20]. Further studies conducted by Mandal et al. [21] concluded that citrus mouthwashes rich in vitamin C were much more effective in reducing gingivitis, a result similar to the current study that proves that the anti-inflammatory effect of *Punica* was efficient in reducing inflammation when compared to *Lawsonia *as it is a rich source of ascorbic acid as reported by Patel et al. [22]. A previous study done by Fernandes et al. concluded that *Punica* can be used as a potent herbal mouthwash attributing to its anti-inflammatory properties. The phenolic compounds present in *Punica* such as epigallic acid and punicalagin were evaluated as follows: (1) inorganic component concentration and reduced demineralization on bovine enamel blocks subjected to pH cycling; (2) anti-biofilm effect on dual biofilms of *Streptococcus mutans* ATCC 25175 and *Candida albicans* ATCC 10231 treated for one and 10 minutes, respectively; and (3) cytotoxicity and production of inflammatory mediators (interleukin 6 and tumor necrosis factor-alpha). They concluded that in the near future, *Punica* can be used as a potent herbal mouthwash, which is in coincidence with the current study as it is also chemical-free [23].

Limitations

The sample size taken was few; better results can be expected with a larger sample size. Long-term follow-up studies can depict better results about the potency of the herbal mouthwashes. Phytochemical analysis along with the structural constituency of each phytoconstituent yields better promising effects about the usage of herbal mouthwashes in fighting inflammation.

Future scope

Herbal mouthwashes can be beneficial over the conventional mouthwashes as it does not cause any side effects such as tooth stains, altered taste sensations, and altered microflora and also as it is alcohol-free; hence, novel herbal mouthwashes can be recommended rather than the conventional chemical mouthwashes available in the market.

## Conclusions

According to the current study, the anti-inflammatory efficacy of *Punica granatum* and *Lawsonia inermis* is attributed to the phytochemical action of compounds found in *Punica* such as gallic acid, epigallic acid, ascorbic acid, anthocyanin, epigallocatechin, catechin, quercetin, and rutin and phytochemicals in *Lawsonia* such as 1,4-naphthoquinone, tannins, flavonoids, terpenoids, xanthones, and saponins. This study demonstrated that *Punica *and *Lawsonia *have potent phytochemicals that fight inflammation. *Punica* is much more effective in cutting down inflammation owing to its productive phytochemicals and can be used as an elective herbal mouthwash.
